# New links between *SOD1* and metabolic dysfunction from a yeast model of amyotrophic lateral sclerosis

**DOI:** 10.1242/jcs.190298

**Published:** 2016-11-01

**Authors:** Emma L. Bastow, Amber R. Peswani, Daniel S. J. Tarrant, Daniel R. Pentland, Xi Chen, Alan Morgan, Gemma L. Staniforth, Jennifer M. Tullet, Michelle L. Rowe, Mark J. Howard, Mick F. Tuite, Campbell W. Gourlay

**Affiliations:** 1 Kent Fungal Group, School of Biosciences, University of Kent, Canterbury, Kent CT2 7NJ, UK; 2 Institute of Translational Medicine, Department of Cellular and Molecular Physiology, University of Liverpool, Liverpool L69 3BX, UK

**Keywords:** ALS, SOD1, Metabolism, Vacuole, Yeast

## Abstract

A number of genes have been linked to familial forms of the fatal motor neuron disease amyotrophic lateral sclerosis (ALS). Over 150 mutations within the gene encoding superoxide dismutase 1 (*SOD1*) have been implicated in ALS, but why such mutations lead to ALS-associated cellular dysfunction is unclear. In this study, we identify how ALS-linked *SOD1* mutations lead to changes in the cellular health of the yeast *Saccharomyces cerevisiae*. We find that it is not the accumulation of aggregates but the loss of Sod1 protein stability that drives cellular dysfunction. The toxic effect of Sod1 instability does not correlate with a loss of mitochondrial function or increased production of reactive oxygen species, but instead prevents acidification of the vacuole, perturbs metabolic regulation and promotes senescence. Central to the toxic gain-of-function seen with the *SOD1* mutants examined was an inability to regulate amino acid biosynthesis. We also report that leucine supplementation results in an improvement in motor function in a *Caenorhabditis* *elegans* model of ALS. Our data suggest that metabolic dysfunction plays an important role in Sod1-mediated toxicity in both the yeast and worm models of ALS.

## Abstract (precis)

**Summary:** In a new yeast model of ALS we have discovered for the first time that mutations in Sod1 can lead to the formation of toxic, soluble proteins that disrupt metabolic regulation.

## INTRODUCTION

Amyotrophic lateral sclerosis (ALS), also known as Lou Gehrig's disease, is a motor neuron disease characterised by progressive muscle wasting. Muscle weakening in ALS patients is caused specifically by the degeneration of motor neurons in the brain and spinal cord (upper and lower motor neurons, respectively) (Waragai, 1997). The majority of ALS cases are sporadic; however, 20% of cases are familial (fALS) and are inherited in an autosomal dominant fashion (Siddique and Hentati, 1995-1996). Mutations in a number of genes have been associated with both sporadic ALS and fALS (Chen et al., 2013), leading to the proposal that ALS is a multifactorial syndrome that includes motor system degeneration. In line with the complex nature of this disease, these genes implicate a range of cellular functions including RNA processing (*TDP43* and *FUS3*) (Arai et al., 2006; Kwiatkowski et al., 2009), cytoskeletal organisation (Dynactin, also known as *DCTN1*, and Profilin, also known as *PFN1*) and vesicle trafficking (*VABP*) (Nishimura et al., 2004). To date, a single drug, the glutamate antagonist riluzole, has been approved for ALS treatment and is reported to lead to a modest extension of median survival of ALS patients (Georgoulopoulou et al., 2013). One of the major challenges is to determine at which stage each of the identified cellular processes contributes to disease progression, and to what extent they represent a target for improved therapeutic intervention.

Up to 20% of fALS cases are associated with a mutation in the *SOD1* gene (Rosen et al., 1993), which encodes superoxide dismutase 1 (Sod1). The Sod1 enzyme is a homodimer that requires copper and zinc binding in order to adopt a stable conformation to facilitate conversion of superoxide anions to hydrogen peroxide and oxygen. Sod1 is primarily cytosolic, but a small proportion is also found within the mitochondrial intermembrane space (Sturtz et al., 2001). The activity of Sod1 relies on Ccs1, a metallo-chaperone that interacts with immature Sod1 to facilitate insertion of a copper ion into the Sod1 active site (Sturtz et al., 2001). When the interaction between immature Sod1 and Ccs1 is disrupted, improper formation of a disulphide bond within Sod1 results in a misfolded form that is prone to aggregation. Human and yeast forms of Sod1 function similarly, yet an important difference between them is that Sod1 activation in yeast is dependent upon Ccs1, but human Sod1 can be activated by other mechanisms (Sturtz et al., 2001).

The aggregation of unstable Sod1 was originally thought to trigger fALS as a result of concurrent loss in Sod1 enzymatic activity (Deng et al., 2006); however, mice lacking the *SOD1* gene do not develop ALS (Reaume et al., 1996) and therefore a toxic gain-of-function is thought to underlie ALS-associated pathology. Over 150 mutations in the *SOD1* gene have been linked to ALS and these are distributed through all five exons. The most common *SOD1* mutation found within the ALS population in the USA is an alanine-to-valine substitution at codon 4 (producing Sod1^A4V^ protein). The A4V mutation is located in the first β-sheet of the protein, at the dimer interface, and affects stability and enzymatic activity (Cudkowicz et al., 1997; Rosen et al., 1994). Reduced stability associated with ALS-linked mutations in Sod1 is generally thought to underpin the toxic gain-of-function. However, it is unclear how each mutation relates to the cellular dysfunction that drives disease progression. Interestingly, the overexpression of native *SOD1* also accelerates disease progression in ALS mouse models (Xu et al., 2015). In addition, the overexpression of *SOD1* alone can lead to ALS symptoms in mice, providing evidence that the native Sod1 protein can participate in disease (Graffmo et al., 2013).

Exactly how *SOD1* mutations trigger or contribute to ALS pathology is unknown; however, the aggregation of misfolded or unstable Sod1 protein is a widely reported hallmark of this disease. Aggregation of Sod1 has been observed in a variety of cell and animal models of ALS as well as within cells and cerebrospinal fluid of ALS patients (Kaur et al., 2016). Sod1 aggregates are reported to accumulate at the outer surface of the mitochondria and within the mitochondrial intermembrane space. The resultant respiratory dysfunction and accumulation of reactive oxygen species (ROS) associated with aggregate accumulation is thought to be an important factor in disease progression (Vehviläinen et al., 2014). In line with mitochondria being a potential therapeutic target, the expression of mitochondrial targeted catalase was sufficient to protect motor neurons from Sod1^G93A^–mediated toxicity in mice (Pehar et al., 2014). However, the neuroprotective effect of catalase was not accompanied by an increase in lifespan, highlighting the complex nature of this disease. The expression of mutant isoforms of Sod1 can also damage a number of other important cellular processes and compartments, including the ER stress response (Soo et al., 2015), glutamate excitotoxicity (Canton et al., 1998), calcium homeostasis (Kawamata et al., 2014), proteasome exhaustion (Kepp, 2015), metal ion regulation (Hilton et al., 2015), inflammation (Van Dyke et al., 2016) and the regulation of autophagy (Mis et al., 2016). It has also become clear that the disease extends beyond motor neurons and affects other cell types such as astrocytes, microglia, oligodendrocytes and muscle cells, whose functions could also play a role in ALS pathology (Kaur et al., 2016). Interestingly, recent studies also suggest that toxic Sod1 protein products exhibit prion-like properties and can spread between cells (Münch et al., 2011; Ayers et al., 2016).

Here, we describe a new model of ALS that we have developed in the yeast *Saccharomyces cerevisiae*. We have incorporated several ALS-linked mutations into the endogenous yeast *SOD1* gene. Our data show that the resulting mutant Sod1 isoforms are unstable and have toxic effects upon the cell. In contrast to other systems studied to date, these mutations do not lead to the formation of Sod1 protein aggregates. In addition, the toxic effects of unstable yeast Sod1 proteins do not appear to cause mitochondrial dysfunction or result in oxidative stress. Instead, our data suggest that toxicity is associated with an inability to control central metabolic processes, most probably linked to severe disruption of the vacuolar compartment. The overall metabolic dysfunction associated with mutant Sod1 isoforms does not result in yeast cell death, but instead drives cells into a state of senescence. Furthermore, in a *Caenorhabditis elegans* ALS model system, in which *SOD1* overexpression results in motor neuron dysfunction, media supplementation with L-leucine rescues motor neuron degeneration. Our findings provide new evidence that soluble, non-aggregating forms of Sod1 might a play role in the cell dysfunction underlying ALS via the disruption of metabolic homeostasis.

## RESULTS

### Expression of yeast *SOD1* ALS mutant alleles is toxic in yeast

**Fig. 1.**
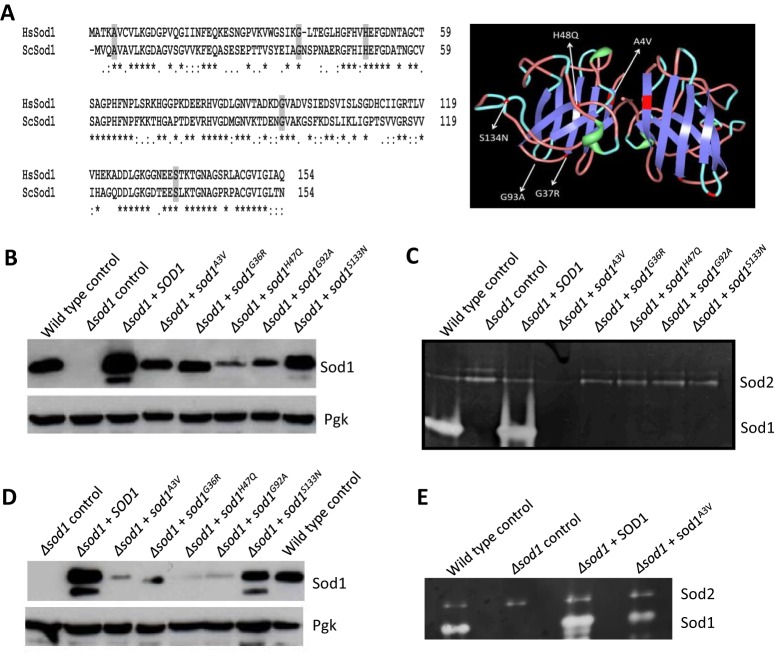
**Yeast *SOD1* ALS mutant alleles**. (A) Amino acid residues commonly substituted within ALS patients and that were mutated in this study are conserved between yeast and human Sod1 (left). Their distribution is indicated upon the crystal structure of a human Sod1 dimer (PDB ID: 1HL4) (right). (B–E) Wild-type or cells deleted for *SOD1* and re-expressing *SOD1* or *sod1* mutants as indicated were assayed for protein level by western blotting during logarithmic (B) and stationary phase (D) of growth or for enzymatic activity during logarithmic (C) or stationary phase (E) of growth.

A number of amino acid residues whose substitutions are associated with fALS are conserved between yeast and humans. To construct a yeast model of ALS we introduced mutations into the yeast *SOD1* gene that led to amino acid substitutions equivalent to A4V, G37R, H48Q, G93A and S134N (Fig. 1A). Each of these mutations are linked with fALS in humans and their expression in mice also gives rise to ALS symptoms (Chen et al., 2013) (Fig. 1A). In yeast Sod1, these mutations correspond to Sod1^A3V^, Sod1^G36R^, Sod1^H47Q^, Sod1^G92A^ and Sod1^S133N^, respectively, and are evenly dispersed over the mature Sod1 protein (Fig. 1A).

To determine the effects of the yeast *SOD1* mutations (called *sod1* mutants hereafter) on cell growth and metabolism, wild-type *SOD1* and the mutants were constitutively expressed from a multicopy plasmid in both wild-type and *sod1*-null (Δ*sod1*) backgrounds, and both protein level and enzymatic activity were assessed. During logarithmic and stationary phases of growth, the overexpression of native *SOD1* could be observed in both wild-type (Fig. S1) and Δ*sod1* strain backgrounds (Fig. 1B,D). However, the level of all five Sod1 mutant isoforms did not exceed, and in most cases was significantly lower, than that of un-mutated Sod1 when expressed in a Δ*sod1* background. This is most probably because of inherent instability within the mutant proteins (Fig. 1B,D). By contrast, the introduction of mutant *sod1* expression plasmids into a wild-type background did result in their overexpression (Fig. S1), indicating that mutant isoforms of Sod1 become stabilised when co-expressed with native Sod1. In line with the production of an unstable product, no Sod1 enzymatic activity could be detected within Δ*sod1* logarithmic phase cells expressing mutant Sod1 isoforms (Fig. 1C); however, low levels of enzymatic activity could be detected in stationary phase cells expressing *sod1^A3V^*, *sod1^G36R^* and *sod1^G92A^* (Fig. 1E and Fig. S2). Levels of Sod2 remained relatively stable in all strains examined (Fig. 1C,E).

**Fig. 2.**
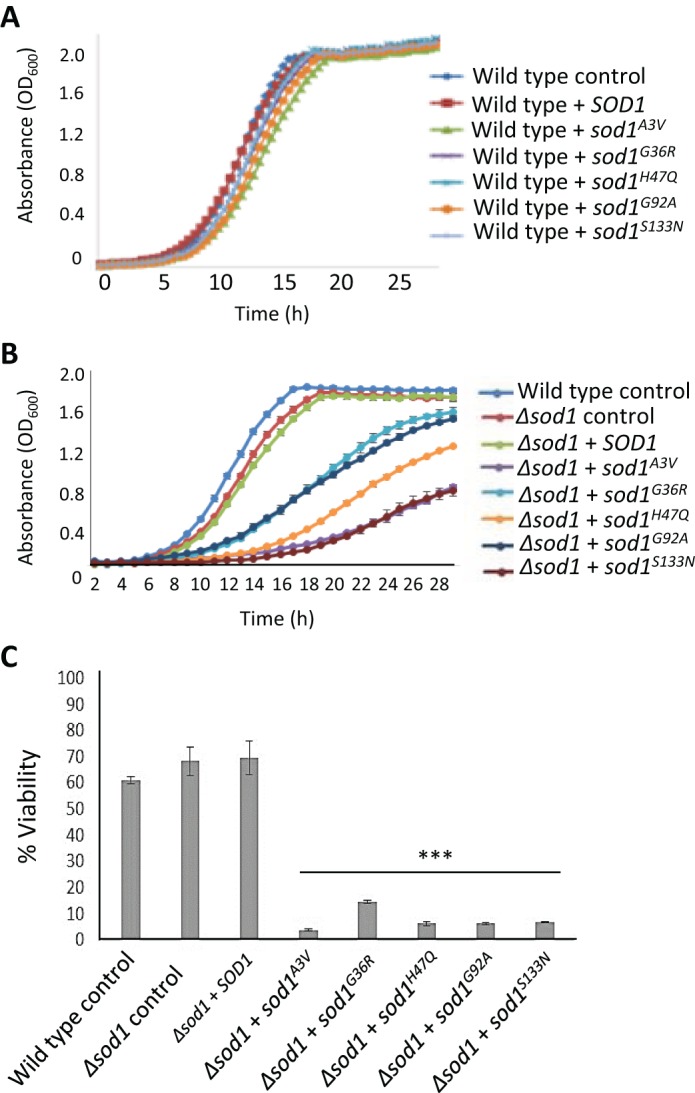
**Effect of *SOD1* mutation on cell growth and viability.** (A,B) Wild-type (WT) or cells deleted for *SOD1* containing either an empty plasmid (control) or re-expressing *SOD1* or *sod1* mutants as indicated were assessed for growth using an automatic plate reader. (C) Viability was assessed after 24 h of growth in selective medium and assessed using a colony forming unit assay, which calculates the percentage of cells able to form a colony within each population. The probability of a significant difference in viability compared with wild type was calculated for each strain; ***P<0.001. All experiments were carried out in biological triplicate and error bars represent standard deviation.

The expression of *sod1^A3V^*, *sod1^G36R^*, *sod1^H47Q^*, *sod1^G92A^* or *sod1^S133N^* had no effect on wild-type cell growth (Fig. 2A) or viability (data not shown). However, expression of the same mutant alleles in a Δ*sod1* strain led to an elongated lag phase, slower growth during logarithmic phase and reduction in final culture cell number (Fig. 2B). Toxic effects associated with the expression of mutant forms of Sod1 also led to a significant loss of culture viability (Fig. 2C). These data suggest a correlation between Sod1 stability and toxicity within our yeast ALS model system. This correlation is also in accordance with our observation that the overexpression of mutant Sod1 isoforms that are stabilised in a wild-type background are not toxic to yeast cells.

### Toxicity associated with Sod1 instability does not correlate with increased ROS levels or mitochondrial dysfunction

**Fig. 3.**
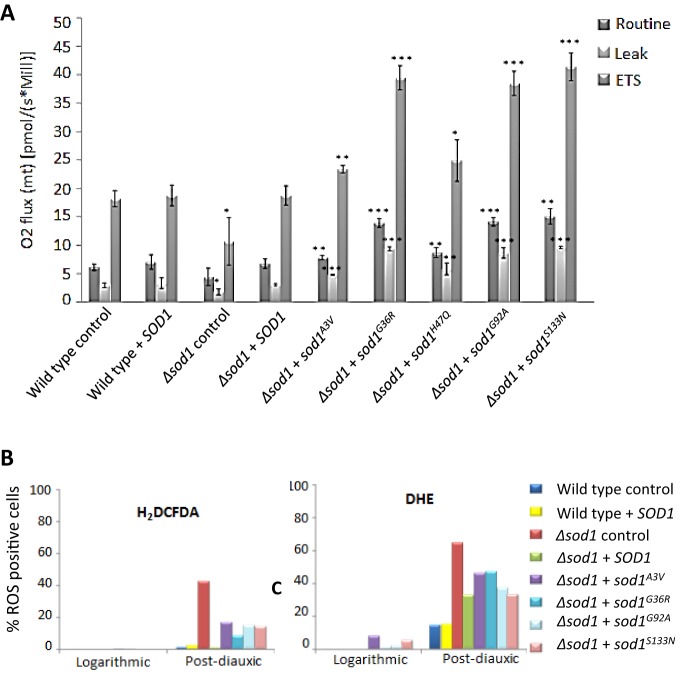
**Effect of *SOD1* mutation on respiratory function and ROS production**. (A) Respiratory function was assessed in wild-type (WT) and Δ*sod1* cells containing either an empty plasmid (control) or re-expressing *SOD1* or *sod1* mutants as indicated by high resolution respirometry. Oxygen consumption was assessed in cells grown for 24 h to stationary phase (Routine) after which TET was added to provide LEAK respiration. FCCP was then added to give maximal (or ETS) respiration levels. Data from biological triplicates is presented and the probability of a significant difference in routine, LEAK or ETS respiration compared with wild type was calculated for each strain; *P<0.1, **P<0.01, ***P<0.001. (B) The production of ROS was assessed by addition of the peroxide sensing dye H_2_DCF-DA or superoxide indicator dihydroethidium (DHE) during both log and post-diauxic phases of growth. Fluorescence was assessed for 10,000 cells in triplicate using flow cytometry. A representative data set is presented. Error bars represent standard deviation.

*SOD1* mutations and their toxic effects have been linked to mitochondrial dysfunction (Vehviläinen et al., 2014). To examine whether this was also the case in our yeast ALS model we determined the respiratory profile of strains expressing *sod1^A3V^*, *sod1^G36R^*, *sod1^H47Q^*, *sod1^G92A^* or *sod1^S133N^* using high resolution respirometry (Fig. 3A). Using this technique, which measures mitochondrial oxygen consumption, the addition of drugs that target specific sites of the electron transport chain (ETC) allowed us to examine mitochondrial function in detail. Initially, routine respiration was analysed in a Δ*sod1* strain expressing *SOD1*, *sod1^A3V^*, *sod1^G36R^*, *sod1^H47Q^*, *sod1^G92A^* or *sod1^S133N^* during the post diauxic shift phase of growth (Fig. 3A). Surprisingly, respiration was significantly increased in all mutant strains relative to wild type (Fig. 3A) despite their low culture viability (Fig. 2C). The increase in respiration observed was consistent with an increase in the activity of the entire ETC, as shown by significant increases in the LEAK respiration rate, which indicates inner mitochondrial integrity and the level of control that ATPase function has on electron transport, and maximum ETC rate (ETS, induced by addition of the uncoupling agent FCCP) (Fig. 3A). The LEAK and ETS oxygen flux in a Δ*sod1* strain were significantly decreased compared with wild-type control but re-expression of *SOD1* returned these to the wild-type control levels (Fig. 3A). The increase in respiration rates observed suggests that the loss of cell viability observed in cells expressing mutant Sod1 is not a result of cell death. To confirm this, Δ*sod1* cells expressing *SOD1*, *sod1^A3V^* or *sod1^G92A^* were grown for 24 h and then plated onto either minimal media lacking leucine to select for plasmid retention (as done previously; see Fig. 2C) or onto rich YPD media (Fig. S3). We observed that Δ*sod1* cells expressing either *sod1^A3V^* or *sod1^G92A^* exhibited a dramatic increase in colony forming units when plated onto rich media instead of minimal selective media (Fig. S3). This finding suggests that the expression of unstable *sod1* isoforms leads to loss of viability caused by senescence that is linked to defects in nutritional sensing or resource availability, which can be rescued by growth in rich media.

Changes in mitochondrial function and a decrease in viability are often associated with ROS production. We therefore assessed the production of both the superoxide anion and hydrogen peroxide during logarithmic and post-diauxic shift phases of growth using the ROS sensor dyes DHE and H_2_DFC-DA, respectively. Significant ROS production could not be detected during logarithmic growth in any of the strains tested (Fig. 3B). As expected, the loss of Sod1 activity correlated with an increase in both superoxide and peroxide levels compared with wild type during the post-diauxic phase (Fig. 3B). ROS levels were also higher in cells expressing *sod1^A3V^*, *sod1^G36R^*, *sod1^H47Q^*, *sod1^G92A^* or *sod1^S133N^*; however, these levels were decreased compared with the Δs*od1* strain. The addition of 1–4 mM of reduced glutathione, which acts as a powerful anti-oxidant, to growth media did not result in a reduction in the toxic effects of expressing *sod1* mutant isoforms in a Δ*sod1* background (data not shown). These findings suggest that the toxicity associated with the expression of ALS-linked *sod1* mutations is not linked to elevated levels of oxidative stress in the yeast ALS model system.

### Toxic yeast Sod1 isoforms do not form insoluble aggregates

**Fig. 4.**
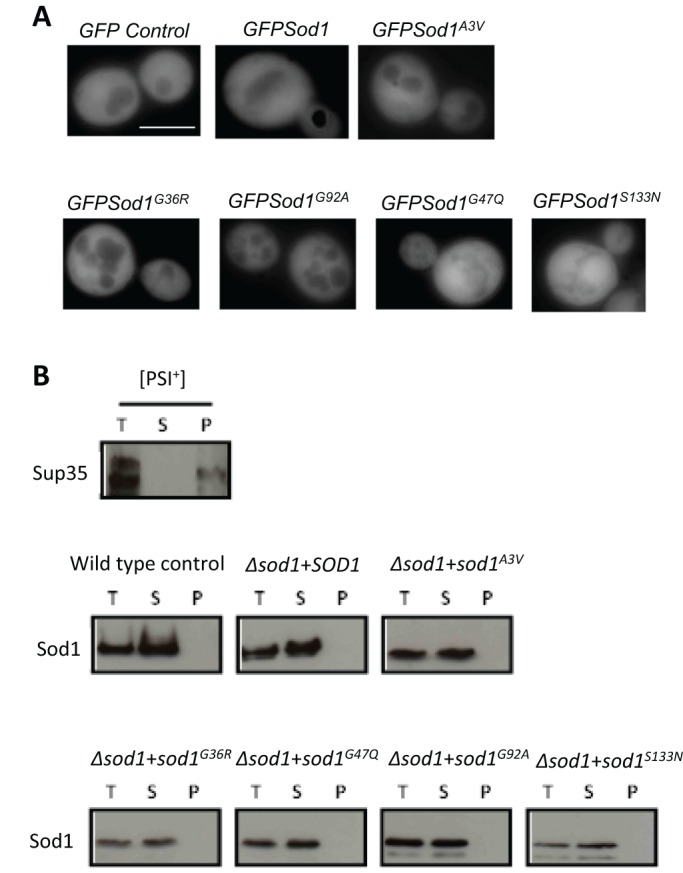
**Aggregation status of Sod1 mutant proteins.** (A) To determine whether mutations in *SOD1* lead to the accumulation of protein aggregates, constructs expressing GFP alone or GFP–Sod1 were expressed in Δ*sod1* cells. (B) The presence of soluble and insoluble Sod1 was assessed in wild-type or Δ*sod1* cells carrying either an empty plasmid (control), *SOD1* or mutant *sod1*, as indicated by sedimentation analysis. Cells expressing the prion-forming protein Sup35p in its *[PSI^+^]* state were used to generate a positive control sample containing insoluble aggregates. Scale bar: 10 µm.

Many studies correlate the toxicity associated with *SOD1* mutations with the appearance of insoluble high molecular weight protein aggregates (Bosco et al., 2010). Our data also suggest that mutations introduce an inherent instability within the Sod1 protein, therefore we sought to determine whether any of the Sod1^A3V^, Sod1^G36R^, Sod1^H47Q^, Sod1^G92A^ or Sod1^S133N^ mutant proteins formed aggregates in yeast. To do this, we expressed GFP-tagged Sod1 proteins in a Δ*sod1* background (Fig. 4A). Although the expression of GFP-tagged Sod1 mutant isoforms was toxic when expressed within Δ*sod1* cells (data not shown), we were unable to detect the presence of GFP foci, indicating a lack of significant aggregate formation (Fig. 4A). To determine whether insoluble SDS-resistant aggregates could be present, but not visible by fluorescence microscopy, we performed a sedimentation analysis. The yeast prion forming protein Sup35 was used as positive control within this assay as it readily forms aggregates when in the [*PSI*^+^] state (Eaglestone et al., 2000). The prion aggregates are found within the pellet fraction after high speed centrifugation (Fig. 4B). Using this analysis, we were unable to detect Sod1^A3V^, Sod1^G36R^, Sod1^H47Q^, Sod1^G92A^ or Sod1^S133N^ aggregates, and found that all of the detectable protein was located within the soluble fraction (Fig. 4B).

### Truncated Sod1 protein can elicit a toxic response

**Fig. 5.**
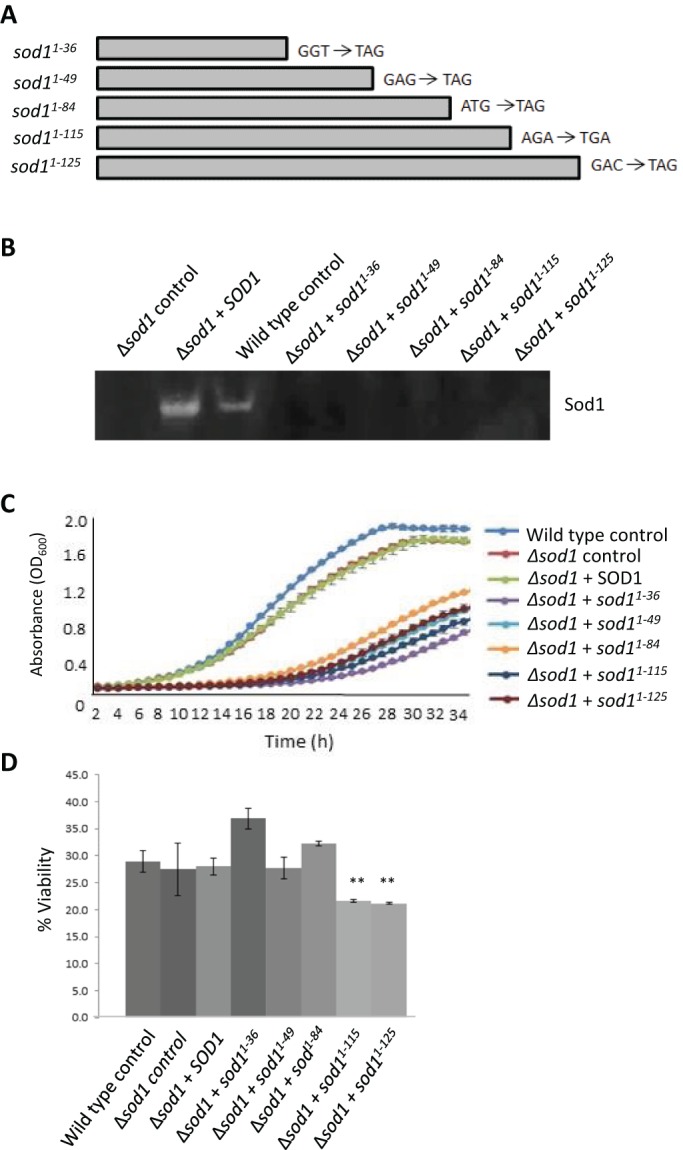
**Effect of truncation of Sod1 on enzymatic activity, cell growth rate and cell viability.** (A) Mutations were introduced into the *SOD1* gene to give rise to stop codons as indicated. (B) The enzymatic activity of these truncated products was assessed on native gels. (C,D) The effects of expression of truncated Sod1 was assessed by growth rate (C) and viability (D) following 24 h of growth using a colony forming unit assay, which calculates the percentage of cells able to form a colony within each population. Experiments were conducted in biological replicates. Error bars represent standard deviation; **P<0.01.

A previous report has suggested that the expression of truncated Sod1 protein can exert toxic effects in a chick spinal cord model system (Ghadge et al., 2006). This promotes the hypothesis that breakdown products of unstable Sod1 proteins may themselves be toxic and so contribute to the ALS condition. To address this possibility in the yeast system, we introduced stop codons into the endogenous *SOD1* gene at regular intervals to allow the expression of truncated Sod1, ranging from 36 to 125 amino acids in length (Fig. 5A). As expected, none of the fragments exhibited enzyme activity (Fig. 5B). Using a GFP tagging approach, we could not detect the aggregation of Sod1 truncated fragments by fluorescence microscopy (data not shown). However, the expression of truncated Sod1 led to a decrease in growth rate in all cases (Fig. 5C). A drop in viability was also observed when truncated Sod1 products of 115 and 125 amino acids were expressed (Fig. 5D). These data lend further support to our observations that the soluble products of unstable Sod1 proteins have deleterious effects on yeast cells.

### Mutant Sod1-associated toxicity is associated with metabolic stress

**Fig. 6.**
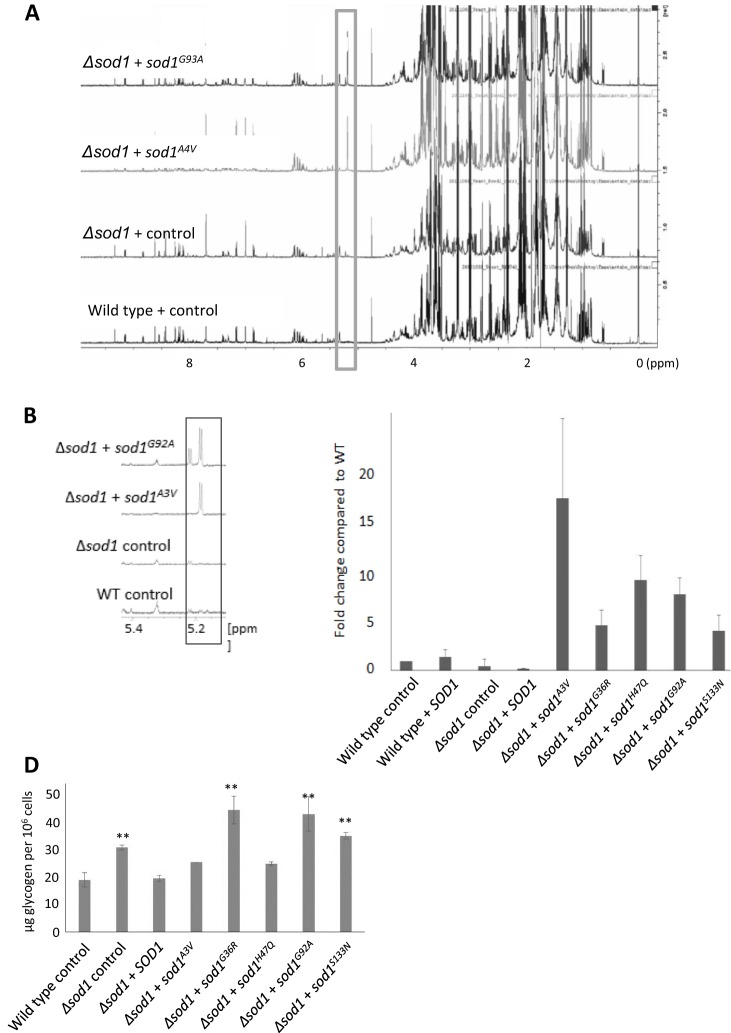
**Association of *SOD1* mutation with metabolic stress.** (A,B) Metabolites were extracted and subjected to NMR analyses. The representative 1D H^+^ spectra display metabolite peaks that highlight the identified trehalose peak, which is enlarged in (B). (C) To confirm the finding, an enzymatic trehalose assay was performed in biological triplicate and the fold change is compared with wild type. (D) Glycogen levels were assessed in triplicate. Error bars represent standard deviation; **P<0.01.

To further examine the effects of ALS-linked *SOD1* mutations on cell health we used an NMR-based approach to produce metabolomic profiles of actively growing wild-type, Δ*sod1*, Δ*sod1*+ *sodA^A3V^* and Δ*sod1*+ *sod1^G92A^* strains. Our analysis revealed a very strong upregulation of trehalose production in cells expressing *sod1^A3V^* or *sod1^G92A^* that was not observed in wild-type or Δ*sod1* cells (Fig. 6A,B and Table 1). This finding was verified using an enzymatic assay and appeared to be a consistent phenotype associated with the expression of any of the Sod1 mutant isoforms tested (Fig. 6C). Glycogen could not be identified by NMR and was therefore assessed by an enzymatic assay. Levels of glycogen were elevated in cells lacking *SOD1* and could be restored to wild-type levels by expression of *SOD1* on a plasmid (Fig. 6D). The levels of glycogen appeared to vary within Δ*sod1* cells expressing mutant Sod1 isoforms, with higher levels found in *sod1^G36R^*, *sod1^G92A^* and *sod1^S133N^* but not *sod1^A3V^* or *sod1^H47Q^* cells (Fig. 6D).

**Table 1.**
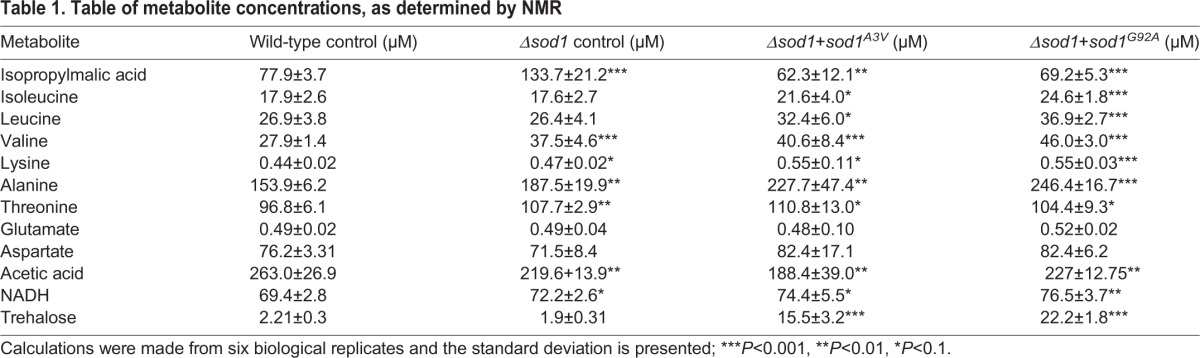
**Table of metabolite concentrations, as determined by NMR**

Our NMR analysis suggested that the levels of a number of amino acids, including leucine, valine, lysine and alanine, were significantly elevated in cells expressing Sod1 mutant isoforms (Table 1) indicating an induction of specific amino acid biosynthesis pathways. This finding is significant because cell samples were analysed in actively growing cultures at a time when amino acids were in plentiful supply. During amino acid starvation the translation of *GCN4* mRNA is increased, which initiates the Gcn4-mediated transcription of amino acid biosynthesis genes (Hinnebusch, 2005). Simultaneously, protein synthesis is inhibited to ensure that cells conserve amino acids. The ability to activate amino acid biosynthesis genes in response to amino acid deprivation therefore provides cells with a survival pathway against starvation.

**Fig. 7.**
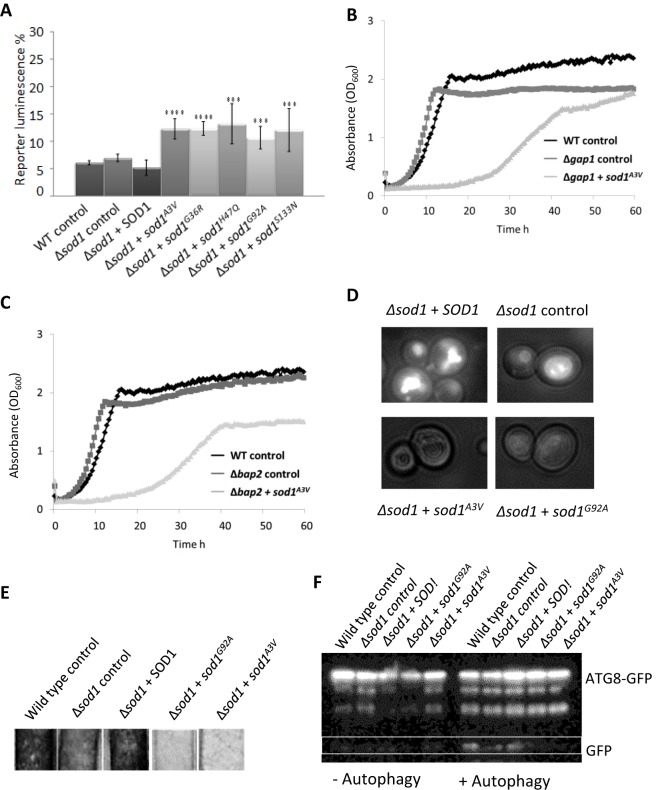
**Effect of SOD1 mutation on amino acid biosynthesis and vacuole function.** (A) Expression of Gcn4p was monitored using a controlled luciferase reporter system in wild-type or Δ*sod1* cells containing an empty plasmid (control), *SOD1* or mutant *sod1* as indicated. (B,C) *sod1^A3V^* was expressed in cells lacking the amino acid permeases GAP1 (B) or BAP1 (C) and the effects monitored by growth analysis. Representative datasets are presented. (D) Quinacrine uptake was used to assess vacuole acidification in cells lacking *SOD1* and containing an empty vector control, or expressing *SOD1*, *sod1^A3V^* or *sod1^G92A^*. (E) H_2_S production was assessed in the indicated strains by incubation with detector strips. (F) Autophagy was assessed in wild type, *Δsod1*+empty control plasmid, *Δsod1+SOD1*, *Δsod1+sod1^G92A^* and *Δsod1+sod1^A3V^* by liberation of free GFP from ATG8–GFP following nitrogen starvation. Error bars represent standard deviation; ***P<0.001.

To assess *GCN4* expression in the various *sod1* mutant-expressing strains, we made use of a luciferase reporter placed upstream of the *GCN4* 5′-UTR. The expression of mutant forms of Sod1 led to a significant increase in reporter activity during logarithmic growth, indicating a strong activation of the general amino acid response (Fig. 7A). Further evidence to support the hypothesis that expression of Sod1 isoforms leads to a failure to regulate amino acid levels was provided by a dramatic synthetic effect when *sod1^A3V^* was expressed in cells lacking the amino acid permease genes *GAP1* or *BAP1* (Fig. 7B and C).

The yeast vacuole, the functional equivalent of the mammalian lysosome, is the main store of free amino acids in cells and is also crucial for the regulation of amino acid levels during times of stress. As a result, defects in vacuole function are associated with failure to regulate amino acid levels. To determine whether expression of mutant *sod1* affects vacuole function, we examined both acidification and morphology of the organelle. A clear defect in the uptake of the dye quinacrine, which is used to assess vacuolar pH (Preston et al., 1989), was observed in cells expressing mutant isoforms of Sod1 (Fig. 7D). A small increase in the number of cells exhibiting multiple vacuoles was also observed for cells lacking *SOD1* and for cells expressing mutant Sod1 isoforms (data not shown). Cells with defective vacuolar ATPase function, which is responsible for acidification of the vacuole, have recently been shown to exhibit decreased H_2_S production (Winter et al., 2014). A clear decrease in H_2_S production was observed in Δ*sod1* strains expressing mutant isoforms of Sod1 compared with controls (Fig. 7E).

Vacuole function is central to the autophagic process, which has been implicated in ALS disease pathology (Sasaki, 2011). To determine whether Sod1 mutation also leads to defects in autophagy we expressed a GFP-tagged version of autophagy-related protein 8 (Atg8) in cells expressing *sod1^G92A^* or *sod1^A3V^*. The hydrolysis of GFP from Atg8 upon autophagy is an established marker for autophagy in response to nitrogen starvation (Huang et al., 2014) and this event can be visualised by western blotting using an anti-GFP antibody. Upon induction of autophagy, free GFP could be detected in wild-type, *Δsod1* and *Δsod1*+*SOD1* cells but not in *Δsod1* strains expressing either *sod1^G92A^* or *sod1^A3V^*, indicating a defect in autophagy (Fig. 7F). Confirming that the process of autophagy is impaired in cells expressing unstable *sod1* isoforms, we found a strong synthetic interaction when *sod1^G92A^* and *sod1^A3V^* were expressed in cells lacking the mitophagy regulator *ATG32* (Fig. S4).

### Addition of L-leucine leads to a decrease in the toxicity of *SOD1* overexpression in motor neurons of *C. elegans*

**Fig. 8.**
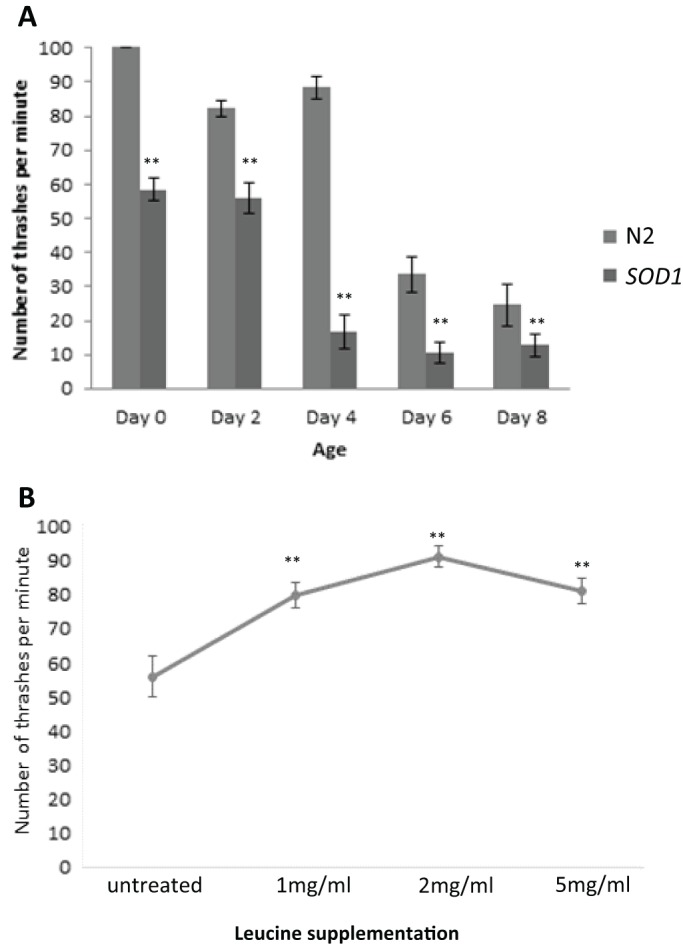
**Effect of L-leucine on toxicity of SOD1 overexpression in *C. elegans*.** (A) A thrashing assay was carried out to assess motor neuron activity in *C. elegans* control (N2) or overexpressing *SOD1* (*SOD1*) over the time course indicated. The thrashing assay was conducted in *C. elegans* overexpressing *SOD1* grown on media containing L-leucine supplemented at 1, 2 or 5 mg/ml as indicated. Error bars represent standard deviation; **P<0.01.

Our data with the yeast ALS model suggest that increased levels of unstable Sod1 can lead to toxicity associated with defects in the regulation of metabolism during growth. The overexpression of native *SOD1* leads to ALS in mouse models (Graffmo et al., 2013), suggesting that elevated levels of Sod1 also lead to a toxic gain-of-function within cells. We therefore used an established thrashing assay to assess whether the overexpression of native *SOD1* in the motor neurons of *C. elegans* led to motor defects. As nematode worms age they lose motor function and their thrashing activity declines over time (Fig. 8A). The overexpression of *SOD1* in motor neurons decreased thrashing activity relative to controls and reproducibly accelerated age-related motor dysfunction (Fig. 8A). Our evidence suggests that amino acid deprivation and the activation of amino acid biosynthesis are important in the toxic effects of mutant Sod1 expression in yeast. Current research suggests that leucine is used to sense amino acid levels and elicit an appropriate response via control of the TORC1 signalling pathway (Ham et al. 2014). Interestingly, we observed that the addition of L-leucine to medium upon which *C. elegans* was growing and feeding gave a dose-dependent and reproducible increase in motor activity in worms overexpressing *SOD1* (Fig. 8B). These data further suggest a link between the toxic gain-of-function associated with Sod1 and the regulation of metabolism in a multicellular ALS model.

## DISCUSSION

Mutations in *SOD1* that are linked to ALS can reduce the stability of the Sod1 protein or homodimer, which in turn promotes aggregation. Such aggregates, which are widely reported within both ALS patient samples and within cell- and animal-based models of the disease, have been suggested to cause dysfunction in a number of cellular compartments (Chen et al., 2013). The overexpression of native *SOD1* has also been shown to accelerate disease progression in mouse models of ALS (Gajowiak et al., 2015). In addition, *SOD1* overexpression alone can lead to ALS-like symptoms in mouse models and has been associated with sporadic cases of ALS. Native *SOD1* overexpression has also been shown to lead to mitochondrial damage (Bosco et al., 2010; Guareschi et al., 2012). Although the aggregation of Sod1 is strongly linked to cellular dysfunction, it remains possible that some of its toxic effects arise from unstable but soluble forms of the protein. The data presented in our study adds weight to this hypothesis because we have identified toxic properties of soluble Sod1 proteins in a newly developed yeast model of ALS.

Although our data support a correlation between Sod1 stability and toxicity, as suggested using other model systems, such toxicity does not correlate with the formation of large aggregates and appears to result from unstable but soluble forms of the protein. It is likely that mutations that destabilise the Sod1 protein lead to structurally altered soluble forms that have deleterious effects on the cell. Within this hypothesis it is also possible that native Sod1 molecules that are not properly folded or incorporated into stable dimers also exhibit toxic effects. This proposal is supported by the fact that overexpression of native Sod1 can lead to disease in mouse models (Gajowiak et al., 2015).

Overexpression of *SOD1* did not lead to measurable effects on cell growth, mitochondrial function or viability in our yeast model system. One possibility is that fragments of the Sod1 protein, generated as a result of proteolysis or instability, represent a toxic species – as suggested in a chick spinal cord model of ALS (Ghadge et al., 2006). This could also be the case in the yeast system, as truncated forms of Sod1 in this study led to defects in growth and decreased viability. Despite observing growth defects as a result of expression of Sod1 truncations of varying sizes, an effect on viability was only observed when the two longest Sod1 (1–115 and 1–125) truncated proteins were expressed. The most severe effects on viability were observed when full-length mutant Sod1 isoforms were expressed, indicating that unstable full-length Sod1 forms are more deleterious to the cell than shorter Sod1 fragments.

Another interesting observation from our data was that overexpression of mutant forms of Sod1 were not toxic in a wild-type background. This suggests that that the presence of native Sod1 is sufficient to stabilise mutant Sod1, which in turn leads to reduced toxicity. Toxic effects of mutant Sod1 could, however, be observed in cells with perturbed mitophagy (via deletion of *ATG32*) or amino acid uptake (via loss of *GAP2* or *BAP2*), despite the presence of endogenous Sod1. This suggests that mutant Sod1 can exert a dominant effect in the yeast system, but that this effect is strongly influenced by genetic background and cell health.

Although the toxic effects of unstable Sod1 expression in cells lacking Sod1 led to a significant loss in viability, this did not appear to be a result of cell death. Instead, our data suggest that mutant Sod1 expression leads to an inability to regulate central metabolic processes, culminating in a senescent phenotype. This manifests in a failure to properly manage carbon during growth, as the biosynthesis of trehalose (a storage carbohydrate associated with the stress response) was significantly elevated in cells expressing *sod1^A3V^* and *sod1^G92A^*. Interestingly it has been shown that trehalose has a strong neuroprotective effect when administered to Sod1^G93A^-expressing mouse cells (Gomes et al., 2010). It has been suggested that neuroprotection occurs as a result of trehalose promotion of the clearance of aggregates via the stimulation of autophagy (Castillo et al., 2013). As we do not observe the accumulation of Sod1 aggregates in our yeast ALS model system, but do observe a significant upregulation of trehalose, it would be of interest to further investigate the role played by this disaccharide.

Of particular interest is our observation that expression of mutant Sod1 isoforms in yeast leads to a striking inability to control amino acid levels during growth. Additional evidence to support defects in amino acid regulation is given by the strong synthetic effect between deletion of the plasma membrane localised amino acid permease genes *GAP1* or *BAP2* and expression of *sod1^A3V^*. An inappropriate reaction to environmental conditions and the activation of amino acid biosynthesis was also suggested by stimulation of the general amino acid response, as assessed by expression of Gcn4. These effects could be a result of Sod1-mediated vacuolar dysfunction. In line with this hypothesis, we observed that cells expressing either *sod1^A3V^* or *sod1^G92A^* were unable to effectively acidify their vacuolar compartments. This finding implies an interaction between soluble toxic Sod1 isoforms and the vacuolar H^+^/ATPase, the protein complex responsible for the acidification process. In line with this, we also observed a significant decrease in the production of H_2_S, a phenotype that has been strongly linked to vacuolar ATPase function (Winter et al., 2014). Further evidence that vacuolar dysfunction occurs as a result of the expression of mutant Sod1 was provided by the observation that these cells also displayed a defect in the process of autophagy, as observed in mouse models of ALS (Mis et al., 2016). Loss of vacuolar function could underpin the metabolic phenotypes observed in our yeast ALS model system because the vacuole is the main amino acid store and is crucial for maintenance of their levels. Failure to regulate or sense amino acid levels is also likely to lead to de-regulation of cell growth signalling systems such as those orchestrated by the vacuole-localised TORC1 complex (Kira et al., 2016). This could offer an explanation as to why the damaging effects of Sod1 on vacuolar function can drive cells towards a senescent state. Sod1 toxicity also appears to involve amino acid metabolism in worms, as shown by the finding that supplementation of the growth media with L-leucine rescued a loss of motor neuron function during ageing of *C. elegans* worms overexpressing *SOD1*.

Interestingly, recent data suggest a strong correlation between the functionality of the lysosome, the mammalian counterpart of the yeast vacuole, and ALS pathology. For example, motor neuron survival is severely compromised upon disruption of autophagy (Ferrucci et al., 2011), a function that centres upon degradation of material within the lysosome. The process of autophagy has also recently been implicated in ALS progression by being attributed to the clearance of aggregates (Sasaki, 2011) and damaged mitochondria (Xie et al., 2015). The lack of clearance of damaged mitochondria in this model may rest upon an interaction between Sod1 and the microtubule transporter dynein, as the overexpression of snapin appears to promote motor neuron survival as a result of its competition with Sod1 for dynein binding in mice expressing human SOD1^G93A^ protein (Xie et al., 2015). However, the knockout of autophagy was not sufficient to cause ALS in mice (Tashiro et al., 2012). In addition, stimulation of autophagy by rapamycin treatment appears to have mixed success, with reports suggesting acceleration of disease (Bhattacharya et al., 2012) and extension of lifespan (Staats et al., 2013) in different models. The role of autophagy in ALS progression and its usefulness as a therapeutic target therefore remains an open question. However, because we also observe autophagy defects in our yeast model of ALS further experimentation may yield important findings on the precise nature of the Sod1–vacuole interaction that could inform future therapeutic strategies.

## MATERIALS AND METHODS

### Strains, plasmids, media and growth conditions

Yeast strains used in this study are listed in Table S1. Unless stated otherwise, cells were grown in a rotary shaker at 30°C in liquid SC-LEU medium (2% glucose, 0.67% yeast nitrogen base without amino acids and 0.164% yeast synthetic complete drop-out medium supplement lacking leucine, obtained from Formedium). The Δ*sod1* mutant was generated using PCR-based gene deletion. Specifically, primers with *SOD1* overhanging sequences (Table S2) were used to generate a *HIS3* marker gene disruption cassette from the pUG27 plasmid as described (Gueldener et al., 2002). The resultant *SOD1* disruption cassette was purified, concentrated and transformed into BY4741 using a standard yeast transformation method. Cells were assayed for growth in 24-well plates using a double orbital shaking frequency of 400 rpm in a FluoStar Optima plate reader (BMG Labtech). The Sod1 antibody used was a kind gift from Valeria Culotta (John Hopkins University) and was raised in rabbit against the *C. elegans* Sod1 protein. Strains were grown overnight in SC-LEU and diluted to an OD_600_ of 0.05 in fresh SC-LEU medium prior to growth analysis. Primers used in this study are listed in Table S2.

### Plasmid construction and site-directed mutagenesis

A gateway donor vector (pDONR222) containing the yeast *SOD1* gene was obtained from Addgene and expression vectors generated using the gateway LR recombination reaction according to the manufacturer's instructions (Invitrogen). All expression plasmids were generated using the pAG425-GPD-ccdB or pAG425-GFP-ccdB destination vectors (Addgene), which contain a 2 µ origin of replication and a constitutive GPD promoter. The plasmids constructed are listed in Table S3. An Agilent Quikchange Lightning Site-Directed Mutagenesis kit was used to generate mutant or truncated *SOD1* expression vectors from pAG425-GPD-*SOD1* according to the manufacturer's instructions. Primers used for mutagenesis of *SOD1* are listed in Table S2.

### High-resolution respirometry

High-resolution respirometry was conducted using an Oroboros oxygraph-2k; Datlab 4 software was used for data acquisition and analysis, providing values for oxygen concentration and respiration (oxygen flux). The exact cell concentration was calculated using a haemocytometer to facilitate an accurate oxygen flux/cell reading. Routine endogenous respiration was assessed before addition of the ATP synthase inhibitor triethyltin bromide (TET) (Sigma-Aldrich) to a concentration of 0.2 mM to yield the LEAK respiration. Following this, the uncoupling agent (FCCP) (Fluka) was added to a final concentration of 12 µM to give the maximal respiration, or ETS. Last, the complex III inhibitor antimycin A (Sigma-Aldrich) was added to a final concentration of 2 µM to provide the non-mitochondrial respiration, which was subtracted from routine, LEAK and ETS values to yield mitochondria-specific respiration profiles.

### Viability and ROS analysis

Viability was determined by plating 600 cells from an overnight culture and analysis of the colony forming units that grew at 30°C in 3–4 days. ROS accumulation was assessed using the indicator dyes H_2_DCFDA or DHE, as previously described (Leadsham et al., 2009).

### Sedimentation analysis of protein extracts

Proteins were prepared by extraction in native cell lysis buffer [10 mM NaPO_4_ pH7.8, 5 mM EDTA, 0.1% (v/v) Triton X-100, 50 mM NaCl, 500 μM PMSF] plus protease inhibitor cocktail (Roche 11836170001) at 4°C by glass bead lysis. The protein concentration in each extract was then adjusted to 300 μg/ml. For sedimentation analysis, a 50 μl aliquot of each protein lysate was transferred to a 7×21 mm polycarbonate tube (Beckman Coulter 343775) that was compatible with a TLA100 rotor. The protein lysates were spun in a Beckman Coulter Optima MAX Ultracentrifuge at 80,000 rpm for 2 h at 4°C. A 30 μl sample of the supernatant was extracted, taking care not to disturb the pellet. This fraction contained soluble (S) proteins. The remaining 20 μl of supernatant was carefully removed and discarded. The pellet (P) was re-suspended in 50 μl of lysis buffer and contained insoluble proteins. The whole cell extract was used as a control for the total protein (T). The sedimentation proteins were then analysed using SDS–PAGE and western blot analysis.

### Trehalose and glycogen level assessment

Strains were grown overnight in SC-LEU medium and re-inoculated to an OD_600_ of 0.1 in fresh SC-LEU medium. Cultures were then grown to an OD_600_ of 0.5 before assessment. For measurement of trehalose levels, 20 OD units (40 ml) of cells were harvested at 1000 ***g*** for 5 min at 4°C, washed in 1 ml of ice cold distilled H_2_O and pelleted at 16,000 ***g*** for 15 s. Cells were then used to determine trehalose or glycogen content. Cells were then incubated for 60 min at 95°C in 250 μl of 0.25 M Na_2_CO_3_, returned to pH 5.2 with 0.15 ml 1 M acetic acid plus 600 μl of 0.2 M sodium acetate. Trehalase (0.05 U/ml) was added to half of the sample and incubated at 37°C overnight. The solutions were then spun at 5000 ***g*** for 3 min and subjected to glucose assay (Sigma GAGO-20) with 10 times less reagent, using method 1 in the manual. Trehalose concentrations were calculated by subtracting the concentration of glucose in the solution without trehalase treatment from the concentration of glucose in the solution with trehalase treatment. For glycogen level assessment, cells were lysed using glass beads in a buffer containing 25 mM citrate pH 4.2 and 2.5 g/l sodium fluoride at 4°C. The homogenate was cleared by centrifugation at 14,000 ***g*** for 5 min and the supernatant used to assess glycogen using the EnzyChrom glycogen assay kit (Bioassay Systems) according to the manufacturer's instructions.

### Hydrogen sulphide assay

Overnight cultures were grown in selective medium with lead acetate strips (Sigma 06728) attached to the lids of each culture vessel. The lids were fastened tightly to ensure that H_2_S was retained in the tube; a dark precipitate indicates the production of H2S.

### NMR metabolomics

Nuclear magnetic resonance (NMR) spectroscopy was used to analyse metabolite extracts. All experiments were carried out at 298 K on a Bruker Avance 3 600 MHz spectrometer, equipped with a QCI-F cryoprobe. Datasets were acquired with 64,000 points and a proton window size of 16 ppm. Spectra were referenced against an internal standard of DSS. The excitation sculpting method was used to suppress the water peak using pulsed field gradients.

Bruker TopSpin™ and AMIX data analysis software were used to analyse the NMR spectroscopy spectrum for each sample. The Madison Metabolomics Consortium Database was also used for the identification and quantification of metabolites using NMR spectroscopy (www.mmcd.nmrfam.wisc.edu/).

### Luciferase assays to measure *GCN4*-luciferase translation

The reporter construct was based on plasmid pTH650 (Chu et al., 2011), which expresses *Renilla* and Firefly luciferases as independent transcripts from a constitutive bidirectional promoter. This plasmid had the *GCN4* 5′-UTR from position −1 to −650 relative to the *GCN4* gene inserted into the *Bam*HI site of this vector, fusing the *GCN4* 5′-UTR to the Firefly luciferase ORF. To assay luciferase activity, cells were grown in 96-well plates and processed as described (Chu et al., 2014).

### ATG8–GFP autophagy assay

Strains containing a low copy plasmid expressing ATG8–GFP (a kind gift from Professor Frank Madeo, University of Graz) were grown in synthetic drop-out medium containing 2% glucose for 24 h to stationary phase. Following this, 2×10^7^ cells were transferred to the same medium, lacking ammonium sulphate to induce autophagy via nitrogen starvation, and incubated at 30°C with shaking for 6 h. Total protein was extracted from cells pre- and post-induction of autophagy and subjected to western blotting using a mouse monoclonal anti-GFP antibody (Sigma 11814460001, 1:1000 dilution) and detected using an anti-mouse IgG conjugated to horseradish peroxidase (Sigma, 1:5000) with a syngene G box Chemi XX6.

### Superoxide dismutase assay

Cells were grown for 24 h to diauxic shift in YPD medium. 2×10^7^ cells were lysed using glass beads in 0.5 ml lysis buffer [10 mM NaPO_4_, pH 7.8, 5 mM EDTA, 0.1% Triton X-100, 50 mM NaCl, 0.5 mM phenylmethylsulfonyl fluoride (PMSF) and Complete EDTA-free Protease Inhibitor cocktail (Roche)]. The assay was carried out as previously described (Wu et al., 2009).

### Quinacrine staining to assess vacuolar acidification

Some 2×10^7^ cells were harvested and washed three times in selective media containing 2% glucose buffered to a pH of 7.5 with 50 mM Na_2_PO_4_ buffer. Cell pellets were re-suspended in 100 µl selective media containing 2% glucose (pH 7.5). Quinacrine was added to a final concentration of 200 µM. Cells were incubated at 30°C for 10 min and then placed on ice. Cells were then washed three times in 500 µl of ice cold wash buffer (50 mM Na_2_PO_4_ pH 7.5, 2% glucose) before visualisation.

### Nematode culture

*C. elegans* were grown under standard conditions on nematode growth media [NGM; 2% (w/v) agar, 0.3% (w/v) NaCl, 0.25% (w/v) peptone, 1 mM CaCl_2_, 5 μg/ml cholesterol, 25 mM KH_2_PO_4_, 1 mM MgSO_4_] agar plates. *Escherichia coli* OP50 was used as a food source. The P*snb-1*::WTSOD-1 strain expressing human SOD1 under control of a pan-neuronal promoter was a generous gift from Dr Jiou Wang (Johns Hopkins University, Baltimore, USA). The wild-type reference strain was Bristol N2 obtained from the Caenorhabditis Genetics Center (CGC, University of Minnesota, USA). All strains were cultured and assays performed at 20°C.

Different amounts of L-leucine (L8000; Sigma) were added to the NGM before autoclaving. Freshly poured plates were stored in the dark at 4°C until 1–2 days before use and then moved to room temperature and seeded with *E. coli* OP50. Animals were grown on the supplemented plates either from birth or L4 stage (day 0 of adulthood). About 10–20 gravid adults of test strains were cultured to lay eggs for 6 h and then removed to set the eggs in synchrony. Plates were inverted and transferred to a container in which descendants were then grown at 20°C. Motility was measured on day 4 of adulthood.

### Nematode thrashing assay

Developmentally synchronised worms were transferred to 50 μl drops of Dent's Ringer solution (10 mM HEPES, 140 mM NaCl, 6 mM KCl, 3 mM CaCl_2_, 1 mM MgCl_2_, pH 7.4) containing 0.1% (w/v) bovine serum albumin to observe their locomotory ability. Thrashing assays were conducted at 20°C. The number of thrashes was counted for 1 min after 10 min of equilibration. A thrash was counted when both the head and tail bent more than 45° away from the anterior–posterior axis and back again. Animals with moving heads and stick-like bodies were scored as partially paralysed but were also analysed for head movements to the left or right of the central body axis (lateral head movement) and for attempts at forward motion (forward advancement). Individuals were categorised as immobilised following 10 s of inactivity. About 10–20 worms per strain were used in each thrashing assay.
